# Potential side effects of toxic and restricted substances found in cosmetics declared to be of natural origin

**DOI:** 10.3389/fpubh.2026.1801090

**Published:** 2026-05-20

**Authors:** Dorottya Koncz, Monika Anne Nemeth, Edina Pallagi, Barbara Tóth, Dezso Csupor

**Affiliations:** 1 Institute of Clinical Pharmacy, University of Szeged, Szeged, Hungary; 2 Institute for Translational Medicine Medical School, University of Pécs, Pécs, Hungary

**Keywords:** BMHCA, cosmetovigilance, EU safety gate, greenwashing, natural cosmetics

## Abstract

**Introduction:**

Cosmetic products are an integral part of daily life. However, although the regulatory framework for such products appears strict, customer safety and product quality are not guaranteed. A large proportion of consumers prefer using cosmetic products that contain natural substances to avoid exposure of their skin to chemicals. Nevertheless, some of the products marketed to meet this need are not natural or even safe.

**Methods:**

This article presents an overview of reports on cosmetic products declared to be of natural origin in the European Union Safety Gate system between 2005 and 2023.

**Results:**

Many reported cosmetic products posed a chemical risk by containing unauthorized substances (64%) or exceedance of authorized limits (14%). Butylphenyl methylpropional (also known as lilial) was the dominant ingredient present in several product categories. Microbial contamination was also detected (12%), but at a markedly lower rate compared to that recorded for the presence of chemicals (79%).

**Conclusion:**

Use of the term “natural” to describe cosmetic products can be misleading because it does not always comply with the criteria for natural and organic cosmetic components. Importantly, such cosmetic products could pose a serious risk to consumers due to hidden unsafe substances. Hence, implementation of appropriate measures is necessary to ensure consumer safety.

## 1 Introduction

Although synthetic compounds are essential components of cosmetics, producers are frequently criticized for using additional substances that may be harmful to human health. The European Union (EU) has banned more ingredients (>1,400) than the United States of America (< 20), and the current regulatory frameworks for skincare products vary between countries (e.g., Canada, Japan, China, and Brazil) (1). Use of dangerous chemicals, such as 1,4-dioxane, formaldehyde, lead/lead acetate, phthalates, or phthalate esters such as dibutyl phthalate, certain fragrance components, hydroquinone, and polyfluoroalkyl substances has been banned or restricted in the EU (2–7). Cosmetics can also contain other hazardous impurities, such as vinyl chloride left in the product during manufacture or residual microbiological contaminants (8, 9). These substances have the potential to cause negative effects on the skin and in the body and may result in various side effects, such as birth defects, congenital abnormalities, diseases such as cancer, allergic reactions, and infertility (10–12). Herbal extracts could offer some cosmetic benefits; however, not all green cosmetics available on the market are entirely natural. Furthermore, occasionally, the phytochemicals utilized in cosmetic formulations have not undergone sufficient scientific scrutiny (13). In some cases, these products are associated with adverse events without any benefit (14).

### 1.1 Regulatory background

In the EU, the European Commission 1223/2009 regulation (15) is the primary regulatory framework for cosmetic products. According to this regulation cosmetic products must be safe for human health under normal or reasonably foreseeable conditions of use. However, Article 3 specifies that, an unintended presence of trace amounts of prohibited substances may be permitted if they arise from impurities, manufacturing, storage, or packaging migration and are technically unavoidable under good manufacturing practice (15, 16). Regulation (EU) 2019/831 amended Annexes II, III and V of Regulation (EC) No 1223/2009 on cosmetic products (17). Article 13 states that cosmetic products must be registered in the Cosmetic Product Notification Portal before they are placed on the market. In addition, this article also covers good manufacturing practices and labeling (18). Labeling plays a crucial role in cosmetovigilance; following the occurrence of unexpected adverse reactions during the use of a cosmetic product, the label is the only point of reference regarding the ingredients of that product (19). The International Nomenclature of Cosmetic Ingredients (INCI) is used globally for labeling according to the chemical composition of the components, thereby helping consumers, healthcare professionals, and manufacturers to uniformly identify compounds (20). Additionally, Regulation (EU) no. 655/2013 of the Commission establishes uniform standards for the justification of claims made concerning cosmetic products (21). Although the regulatory framework established by the EU is robust, its effective implementation in practice often relies on the awareness and compliance-oriented approach of manufacturers and distributors. Several certification bodies are attempting to counterbalance manufacturers who are selling products that often merely mimic natural origin (greenwashing); however, ultimately, labeling depends on the manufacturer (22). In conclusion, natural certification is not mandatory for these products.

The Safety Gate is the rapid alert system for dangerous non-food products of the EU, based on the Commission's Implementation Decision (EU) 2019/417 of November 8, 2018, which sets the basic principles for the management of the Rapid Exchange of Information System (RAPEX). This enables national authorities to exchange information regarding dangerous non-food and non-pharmaceutical products posing a potential threat to public health within the 30 participating countries of the Safety (23). Gate network (EU Member States, Iceland, Liechtenstein, and Norway) (22, 23). The Cosmetic Ingredient Review (CIR) program is a well-established safety evaluation framework. where scientific staff compile and summarize available evidence (for example through literature search), while independent expert panels perform the final safety evaluation and issue final safety assessment monographs. This process is crucial, because non-compliance does not necessarily indicate a direct risk to consumer health, as toxicological risk depends on exposure conditions and other parameters (16, 93).

### 1.2 Criteria for natural and organic cosmetic products

At present, there are several agencies with different standards. Consequently, determining the most appropriate certification for verifying the natural origin of products can be challenging for manufacturers (24). The COSMetic Organic and Natural Standard (COSMOS) is a harmonized framework involving five European agencies, namely Bundesverband Deutscher Industrie-und Handelsunternehmen, Cosmebio, ECOCERT, Institute of Certification for Ethics and the Environment, and Soil Association (24), aiming to standardize recommendations. Under the COSMOS standard a cosmetic can be natural “COSMOS natural product” and organic “COSMOS organic” product. In both categories, the presence of organically farmed ingredients is a prerequisite; nonetheless, for a product to be labeled organic and natural, it must contain a higher proportion of organically produced ingredients. A beauty care product is certified only as “COSMOS organic product” if ≥95% of the plants it contains are organic and ≥20% of organic ingredients are present in the total formula (10% for rinse-off products) (25, 26). Certification bodies such as ECOCERT certify compliance with the COSMOS standard and may issue “COSMOS natural” certification for products meeting the defined criteria (e.g., a high proportion of ingredients of natural origin) (25, 27). Several certification bodies have a positive list of ingredients for their accepted raw materials, which help navigate between requirements (25).

Notably, although the products we evaluated in the present study may not have been clearly labeled as COSMOS organic or COSMOS natural products, their product names and/or brand names suggested a natural origin. From a total of 2,561 notifications, additional cosmetic products suggesting natural origin could have been selected for our analysis if the images attached to the notifications had been reviewed individually, as the style of the packaging itself may suggest natural origin. However, due to the large number of notifications and their subjective assessment, their inclusion in this analysis was not feasible.

## 2 Materials and methods

### 2.1 Search strategy

We accessed all possible reports from 2005 to 2023 related to cosmetic products to provide initial signals of potential greenwashing-related issues, based exclusively on notifications available in the Safety Gate system. We conducted a complete search in the Safety Gate database and set the filter of the product category to “cosmetics” (9). Products were selected based on their associative link to naturalness, as inferred from their naming or marketing claims. The first search was conducted on October 17, 2023, and was repeated on January 1, 2024 to cover the whole year. The data were exported in xls format and further analyzed. Each year was exported and scrutinized separately. It is important to clarify that substances were not predefined as part of the search strategy; rather, they were identified and compiled based on the results retrieved through keyword-based searches.

### 2.2 Inclusion criteria

#### 2.2.1 Selected reports

According to the main concept, we filtered the results by the most common words, which usually refer to natural origin. Verbatim words were “natur” for natural, “botan” for botanical, “herb” for herbal, “organ” for organic,” “phyto,” “fito,” “bio,” “green,” “plant,” “vegan,” and/or “eco.” During the analysis, the set of relevant terms was iteratively expanded; following each refinement, the entire dataset was re-screened to ensure consistency. Every row with possible coincidences was accessed, namely product, name, description, brand, and packaging description. The substances were accessed in the risk column. We manually added the following: substance, type of risk, product category, and reason for deletion columns. The records were analyzed, and the problem and/or problematic ingredients were collected separately in a different row. One report may contain more than one problematic issue.

#### 2.2.2 Justifications

Results containing only partial word matches without referring to natural origin or in contexts clearly unrelated to natural origin were excluded from our search (e.g., when the phrase “eco” only referred to eco-friendly, such as ecodeodorants, it was excluded from our search). The mere presence of a plant species name was not considered sufficient for inclusion in the analysis. The “Package Description” column was also inspected. Where the association with the study scope was not clear within one column, inclusion decisions were based on multiple data fields.

### 2.3 Categorization of risks and product types

#### 2.3.1 Classification of substances

Regulation (EC) No 1223/2009 on cosmetics was accessed to obtain all ingredients in the most appropriate category, where Annex II contained the prohibited substances in the EU and Annex III contained ingredients that can be used only with restrictions (15). It should be noted that Regulation (EU) 2019/831 amended the Cosmetics Regulation 1223/2009 (17). It is also important to distinguish between ingredients and impurities. Substances intentionally added to the product are considered ingredients, whereas unintentionally present substances are regarded as impurities (16).

Based on the quality issue of the substance within a report and the current regulation, we subgrouped the ingredients into five main categories. Category (1) (unauthorized ingredients) contained substances listed in Annex II that were intentionally used as ingredients.

The category (2) (level quality issue) was created for substances listed in Annex III, typically preservatives, where regulatory limits were exceeded.

The microbial contamination Category (3) was created for impurities of various bacterial and fungal species.

Heavy metal contamination (category 4) was generally classified as impurity; however, in certain cases (e.g., skin-lightening products), they may have been intentionally added as ingredients.

Finally, Category (5) consisted of other cases related to non-compliance with legal requirements, including inadequate labeling (Supplementary Figure S1).

#### 2.3.2 Classification of products

Product were classified based on their application. Hair products (1) included products intended to be used on the hair and scalp. Showering products (2) included bath foams, shower gels, and soaps. The perfume category (3) included eau de toilettes and any types of perfume. Other products were analyzed separately (4). Special creams (5) included peeling creams, massage creams, or creams intended for the face or around the eyes. Skin-lightening products represented a separate subgroup (6).

#### 2.3.3 Other categorizations

Type of alert (seriousness) and counterfeit data were also accessed. Moreover, we searched for the country of origin of the products and the country of submission within the examined reports.

### 2.4 Statistical tools

We used Microsoft Excel (Microsoft Corp., Redmond, WA, USA) to analyze data of illegal cosmetic products that were falsely described as natural. Pivot tables and diagrams were made to analyze and represent the data. Data visualization was additionally performed using R (version 4.5.2; R Foundation for Statistical Computing, Vienna, Austria). The descriptions of the reports at the EU Safety Gate became more detailed and of higher quality from year to year.

### 2.5 Bias

When interpreting the results, it should be taken into consideration that the analysis is based exclusively on the Safety Gate database (does not include other data sources like CIR, SCCNFP, SCCP, SCCS) and therefore does not reflect the actual prevalence of problematic products on the market. Another limitation of the study is that the associative, nomenclature-based search introduces subjectivity and potential selection bias. This study should be interpreted as indicative signal for further safety evaluations and serving as a starting point for independent expert evaluations for identifying potential greenwashing issues within the European Union. As the Safety Gate system relies on notifications from national authorities, observed differences between countries may reflect variations in national reporting practices.

## 3 Results

Overall, 2,561 reports were accessed in the Safety Gate database from 2005 to 2023, containing 289 reports/324 substances with the key words referring to natural origin. From 2005 through 2006, there were no reports matching our criteria. The number of reports increased annually (Supplementary Table S1). All reports accessed were classified as serious risk types.

**Figure 1:**
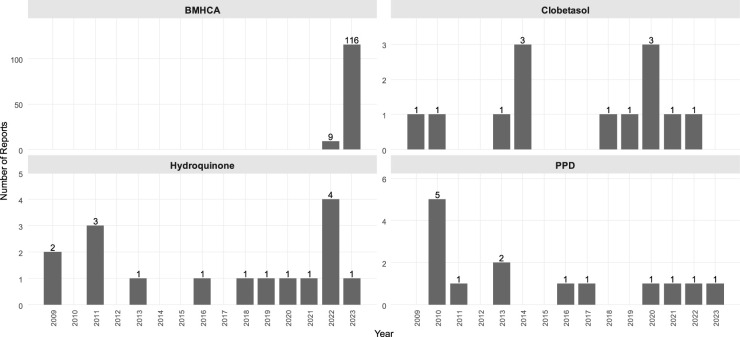
Notifications on the most common ingredients with quality issues in allegedly natural products based on the EU Safety Gate (2009–2023). ^*^BMHCA = butylphenylmethylpropional syn. Lilial; PPD, paraphenylenediamine; Clobetasol, Clobetasol propionate.

**Figure 2:**
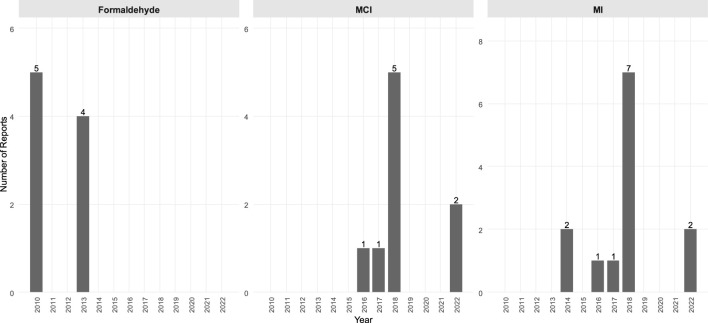
Notifications on the most common preservatives in the EU Safety Gate in allegedly natural products (2010–2022). MCI/MI, Methylchloroisothiazolinone/methylisothiazolinone.

**Figure 3:**
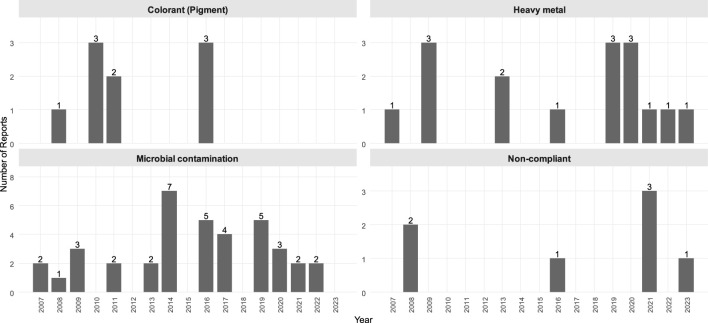
Notifications on the most common subgroups in the EU Safety Gate in allegedly natural products (2007–2023). Not compliant, Inadequate labeling.

Based on our systematic review of the Safety Gate reports in connection with cosmetics, the most common synthetic ingredients were butylphenyl methylpropional (BMHCA; also known as lilial) (125 reports), clobetasol propionate (13 reports), formaldehyde (9 reports), hydroquinone (16 reports), paraphenylenediamine (PPD; 14 reports), methylchloroisothiazolinone [or 5-chloro-2-methyl-4-isothiazolinone (MCI); nine reports] and/or methylisothiazolinone [or 2-methyl-4-isothiazolinone (MI); 13 reports]. There were records of different heavy metal residuals (18), bacterial or fungal-originating microbial contaminations (28), different banned colorants (11) or other discrepancies with the law (7) (Figures 1–3, Supplementary Table S2). The most common problematic product types were hair products (28%), perfumes (21%), skin-lightening applicants (13%), creams (12 %), and different showering products (6%); other categories of problematic products included dental hygiene products, sunscreens, and deodorants (Supplementary Figure S2, Supplementary Table S3).

**Figure 4:**
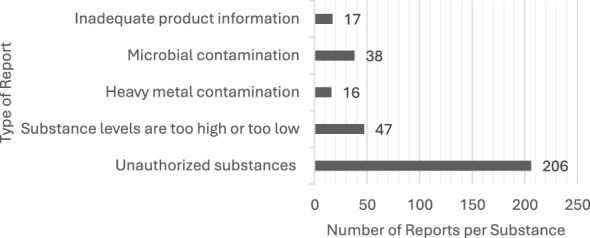
Distribution of the types of reports of non-compliant cosmetic substances (324/289 products).

**Figure 5:**
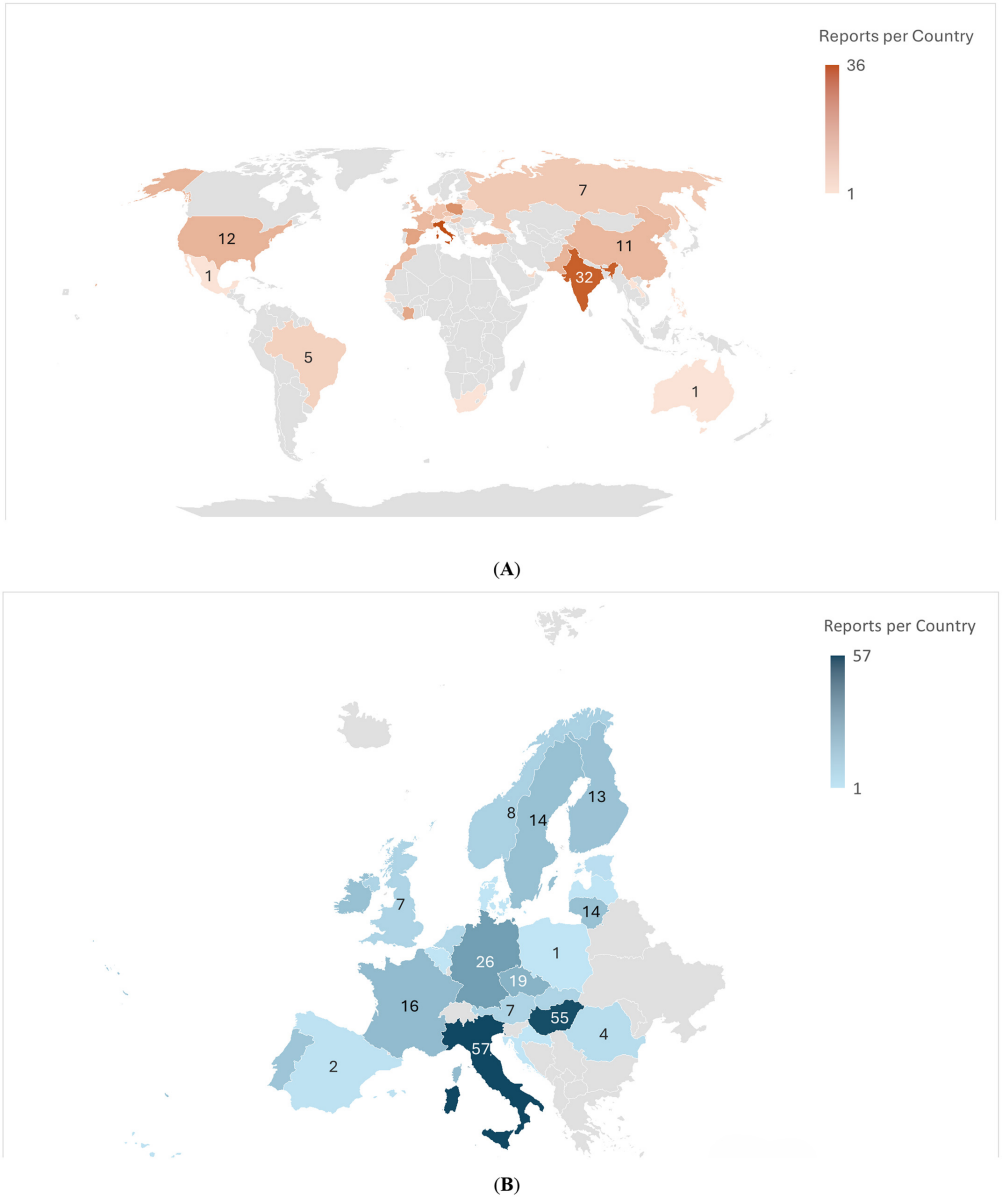
**(A)** Countries of origin of the products: reports per country. **(B)** Countries where the products were notified in Safety Gate: reports per country.

The most common problem with reported cosmetics was the presence of unauthorized substances (64%), followed by the presence of inappropriate substances or exceeding the maximum allowed amounts (14%). The third most common threat was the presence of microbial contaminants (12%). Heavy metal contamination (5%) and other non-compliance (as a category) such as incorrect labeling were present in a smaller amount of products (5%) (Figure 4). While problematic products are present worldwide (Figure 5A), our study was limited to countries which are part of the Safety Gate system (Figure 5B). The highest number of reports was recorded in Italy (*n* = 57), followed by Hungary (*n* = 55). Most of the contaminated products originated from Italy (*n* = 36) and India (*n* = 32). Overall, 131/289 of the products originated outside the EU, European Economic Area member states, and the United Kingdom. Additionally, 21 reports were of unknown origin (Supplementary Table S4).

There were different typical quality problems in each product category. BMHCA (25%) and microbiological contamination (17%) were the two most common contaminants found in hair products (*n* = 82). These products also contained PPD (15%), formaldehyde (9%), MCI and MI (3%−3%), unauthorized colorants (6%), and 2-phenoxyethanol (2%) (Supplementary Figure S3). In addition, 84% of the perfumes contained banned BMHCA (Supplementary Figure S4). BMHCA, MI, MCI, microbial contamination, and heavy metals were present in creams (28%, 20%, 13%, 10%, and 8%, respectively) (Supplementary Figure S5). The most common quality issue in skin-lightening products was the presence of hydroquinone (38%), followed by the presence of clobetasol (31%), heavy metal contamination (20%), and the presence of kojic acid (8%) (Supplementary Figure S6). BMHCA was the most commonly reported synthetic substance in shower products (84%) (Supplementary Figure S7). Microbial contamination was the dominant quality issue among natural dental hygiene products (46%), followed by the presence of diethylene glycol (18%) (Supplementary Figure S8). For sunscreens, 71% of issues were related to labeling, because the claimed sun protection factor (SPF) did not reflect the actual SPF value. Other problematic products with few reports (< 8 products/category) include skin peeling products, makeup and makeup remover products, foot balms, facial toners, face masks, eye balms, detergents, deodorants, black cosmetic clays, bath foams, baby wipes, and aftershaves (Supplementary Figure S9). The products reported had a column indicating whether or not they were counterfeit; however, this could not be clearly established (31% were not marked as counterfeit, while the rest were marked as unknown).

## 4 Discussion

### 4.1 Chemical risk

Although the products under examination seemed to have some natural characteristics, the primary hazard identified in this analysis was the presence of unauthorized chemical compounds. In products described as “natural,” it would have been logical to expect excessive microbial presence rather than hazardous chemicals. However, the results of this study revealed that a large proportion of products contain inappropriate chemicals, indicating that the labeling of these products is consciously misleading. In the following sections, we discuss the three product categories with greatest numbers of quality issues (i.e., perfumes, skin whitening products, and hair products) and the most hazardous active substances within them.

#### 4.1.1 Perfumes

##### 4.1.1.1 Butylphenyl methylpropional

The most frequent threat in perfumes was the presence of BMHCA (84%). BMHCA is a fragrance ingredient, which only started to appear in the Safety Gate in 2022 (69/212 reports). This substance was used in numerous household and personal care products due to its close similarity to the smell of the fresh “lilly of the valley” flower (29, 30). Since March 2022, the European Commission prohibited its use in cosmetics because of its potential to harm the reproductive system, including the health of the fetus, and cause skin irritation (31). BMHCA was the subject of 1,012 of 1,098 (92%) notifications in 2023, denoting a significant difference compared to the rates reported in other years (Supplementary Table S1). Not surprisingly, BMHCA was most frequently found in perfumes, but was also present in shower products, hair products, creams, sunscreens, deodorants, and detergents. Potential toxic effects may be related to the conjugation of *p*-tert-butylbenzoic acid (a metabolite of BMHCA) with coenzyme A, which affects lipid metabolism (30, 32). The increasing number of notifications reported in 2023 was mainly due to the increasing number of BMHCA-related notifications.

##### 4.1.1.2 Other aromatic compounds

Additionally, several fragrances with allergenic potential such as linalool, geraniol, limonene, and eugenol also appeared at elevated concentrations in perfumes and dental hygiene products. Although these compounds can be found in plants, they are potential sensitizers and allergens. Therefore, it is crucial to include these ingredients in the label of products so that customers are aware of their potential for irritation and allergies. However, these materials are often natural components of some essential oils (e.g., essential oils derived from *Citrus* spp.). Directive 2003/15/EC specifies that if the proportion of these substances exceeds 0.01% for rinse-off products and 0.001% for leave-on products, it must be disclosed on the label (19, 33).

#### 4.1.2 Skin-lightening products

The use of toxic ingredients in skin-lightening products has a long history in the cosmetic industry (34). There is a high demand for skin-lightening products, as theoretically they might be used for any type of pigment reduction purposes (e.g., freckles and hyperpigmentation) (35). However, according to a study that focused specifically on substandard skin-lightening cosmetics between 2005 and 2018, this category represented 26.3% of all low-quality cosmetic products in the EU (35).

##### 4.1.2.1 Hydroquinone (benzene-1,4-diol)

Hydroquinone (benzene-1,4-diol) is a phenol derivative that inhibits the tyrosinase enzyme; because of this activity, it is used in the pharmaceutical industry (36, 37). Hydroquinone, applied at concentrations between 2% and 5%, produces visible pigment-reducing effect after only 5–7 weeks (38, 39). In case of clinicl application, a course of treatment lasts at least 3 months and should be discontinued within 1 year (36, 39). Hydroquinone has previously been described as a carcinogenic and hepatotoxic agent (28, 40). According to Regulation (EC) No. 1223/2009 of the European Parliament and of the Council (Annex III), the maximum concentration of hydroquinone in ready-for-use coloring agents for artificial nail systems is 0.02%. Moreover, such products must contain the warning “for professional use only” and “contains hydroquinone,” among other warnings (15). Because of its potentially toxic effects, its use in cosmetic creams is prohibited.

##### 4.1.2.2 Clobetasol propionate

Clobetasol propionate is a synthetic glucocorticoid frequently used in the treatment of inflammatory skin conditions (e.g., psoriasis and eczema), and is typically available as a prescription-only medicine (41–43). Uncontrolled usage of clobetasol propionate as a topical skin-lightening remedy has been reported in the United Kingdom (44). Although the use of clobetasol may offer short-term benefits, it could later have major negative effects. Clobetasol causes local vasoconstriction, resulting in the illusion of immediate lightening of the skin and reduction of pigmentation within the first couple of days of application (45). Thereafter, serious local adverse side reactions can manifest, most frequently on the face, including inflammatory perioral acne (44). The main problem is the inappropriate use and abuse of topical steroids, which can cause severe skin damage, such as striae, atrophy, tinea incognita, erythema, and visible veins (46). According to Regulation (EC) No. 1223/2009 (Annex II), the use of corticosteroids, including clobetasol, in cosmetics is forbidden and illegal.

##### 4.1.2.3 Kojic acid

The prresence of kojic acid (5-hydroxy-2-hydroxymethyl-4-pyrone), a tyrosinase inhibitor and potent antioxidant, was also reported in some products (8%) (47). According to the Cosmeceutical Ingredient Review, this compound can be cytotoxic at concentrations >1% (48). Its use is associated with the occurrence of dermatitis, as well as irritation and sensitization of the skin (49).

This compound is not prohibited in the EU and can be used as a skin whitening agent; however, its concentration exceeded the safe level in certain products (50). Its concentration in ready-for-use preparations should be no more than 1% (15).

##### 4.1.2.4 Mercury

The relatively high rate (20%) for the presence of mercury in skin-lightening products is concerning. The presence of this heavy metal can most probably be attributed to its pigment-reducing effect; however, use of mercury in cosmetics is forbidden due to its toxic effects (15, 51). Our results highlight that skin-lightening cosmetic products tend to contain mercury, and this tendency was observed worldwide (52). According to Annex V of the Cosmetic Products Regulation, phenylmercuric salts (e.g., phenyl mercuric acetate) or thiomersal might be used as preservatives in certain cosmetic products (eye product), but the maximum concentration of mercury is restricted to 70 ppm, and proper labeling is also required (15). Symptoms arising from mercury poisoning, specifically from skin-lightening creams, can include skin rashes, kidney damage, tremors, and severe pain (53). Since mercury is absorbed through the skin, topical use might also be associated with systemic side effects (54, 55). Furthermore, mercury poisoning is not always recognized because of its non-specific symptoms (56).

Plant extracts, such as liquorice, green tea, soy and its extracts, mulberry, and coffee berry, can potentially be a safe and effective solution for this issue; nonetheless, it is important to assess their overall risk-benefit ratio (55, 57). According to a study, *Perilla frutescens* extract significantly decreases the melanin index over a period of 4 weeks without any cytotoxicity (58).

#### 4.1.3 Hair products

Although they are most commonly rinse-off cosmetics, based on their coloring effects, hair products can be permanent or non-permanent.

##### 4.1.3.1 *Para*-phenylenediamine

*Para*-phenylenediamine (PPD) (also known as 4-paraphenylenediamine; 1,4-diaminobenzene, or 1,4-phenylenediamine) is a widely used ingredient in permanent hair dye products (59). The PPD derivative 2-nitro-1,4-phenylenediamine, which is also frequently found in hair colorants, was handled in our study together with PPD (60, 61). PPD used to be present in most permanent hair dye products, but was later found to result in skin responses (e.g., allergic contact dermatitis, erythema) and affect the face, scalp, or ears (62). The process by which PPD produces the black color depends on a secondary element, such as an oxidant or developer. PPD oxidizes to an allergenic hapten inside the epidermis or dermis (62, 63). In the case of PPD, labeling (including the level of the ingredient) is of key importance. According to Regulation (EC) No. 1223/2009, it can be present at a concentration up to 6% (as a free base, not for eyelashes or eyebrows) only as an oxidizing coloring agent in hair dyes. In addition, warnings that the product should be used mainly by professionals or cause allergic reactions must be included in the label (15). Excluding those listed in Annex II, *N*-substituted derivatives of p-phenylenediamine and their salts are permitted for use in hair dye products (15).

##### 4.1.3.2 Colorants (pigments)

Colorants are listed based on their Color Index (CI) numbers in several industries, including cosmetics (64). Seventeen hair dyes, including 12 which could have been used in the past in permanent hair dye formulations, have been banned by Annex II (15). Use of these colorants (pigments) is mainly restricted due to a lack of safety information (65). Our analysis identified several colorants that are banned in the EU [e.g., colors such as violet CI 16036; CI 11154 (INCI name-Basic Blue 41); CI 51319 (Violet 23); CI 20170-2011, CI 41090; Solvent Red 23 (CI 26100); Solvent Yellow 33 (CI 47000); and Solvent Green 3 (CI 61565)] mainly in hair products. Additionally, although colorants used in skincare and hair products may be derived from a natural source, they can be contaminated with heavy metals such as lead, cadmium, and mercury (66). This is commonly observed for certain colorants (e.g., mica) which are mined from areas contaminated with heavy metals (67). Annex IV lists the colorants permitted in cosmetic products in the EU (15).

##### 4.1.3.3 Aminophenols

Aminophenols, such as *ortho*-aminophenol (*o*-aminophenol), were also present in some reported products. According to updated Regulation (EC) No 1223/2009, *o*-aminophenol is prohibited (Annex II), whereas *p*-aminophenol is restricted (Annex III). The latter is permitted for use in oxidative hair dye products and eyelash coloring products under specific conditions, with a maximum concentration of 0.9% after mixing and subject to professional-use and warning requirements. The number of reports was low (*n* = 5, 5%), and were identified exclusively within hair products. These compounds have the potential to cause contact dermatitis when used at higher concentrations (68).

##### 4.1.3.4 Formaldehyde

Free formaldehyde is one of the most effective chemicals for preserving cosmetic items due to its bactericidal and fungicidal qualities (69). Notably, formaldehyde can be found in several naturally occurring sources, including fruits and vegetables (70). Commission Regulation (EU) 2019/831 amended Regulation (EC) No 1223/2009, banning the use of formaldehyde as a preservative in cosmetic products (17). In addition, Regulation (EU) 2022/1181 introduced updated labeling requirements for formaldehyde-releasing substances, mandating the label “contains formaldehyde” when the concentration in the finished product exceeds 0.05% (71). Many personal care items, particularly makeup products, include formaldehyde-releasing preservatives, such as dimethyloldimethyl hydantoin (69). Formaldehyde can provoke dermatitis. Moreover, since it induces the creation of crosslinks between the nitrogenous bases of DNA nucleotides, it can possibly cause DNA mutations; therefore, it is considered a potential carcinogen (72).

##### 4.1.3.5 5-Chloro-2-methyl-4-isothiazolinone and 2-methyl-4-isothiazolinone

MCI and MI are common preservatives often used in combination, particularly in products which are difficult to preserve, such as those stored in a humid environment (73). Multiple cases of allergic contact dermatitis reactions in epidemiological studies linked to the use of MI and MCI were reported (74). Their sensitizing effect led to the ban of MCI/MI for use in leave-on products (but not in rinse-off products) in the EU as of 2016 (75). In rinse-off products, the maximum concentration allowed is 0.0015% (of a mixture in the ratio 3:1 of MCI and MI).

##### 4.1.3.6 2-Phenoxyethanol

2-Phenoxyethanol is used as a preservative and considered a rare sensitizer that can be safely used at concentrations up to 1% (76). It is also found in nature in sources such as green tea (77, 78). In our analysis, we detected a few instances in which the concentration of this preservative exceeded the permitted limits (2%). According to a recent safety review, adverse events associated with 2-phenoxyethanol in animal toxicological studies occurred only when the exposure levels were significantly higher than those typically linked to the use of cosmetic products by customers (76). Other product categories (makeup, deodorants, aftershaves, etc.) were also affected by chemical risks; however, their occurrence was less dominant in these categories (Supplementary Figure S9).

### 4.2 Other quality risks

5% of the reports could not be further categorized based on specific contaminants or specific active substances, so they were classified as non-compliant. These mainly included sunscreens with inadequate SPF labeling (71%). According to the 2006/647/EC principle, the SPF has to be equal to or exceed the value stated in the label (79). It is important to emphasize that the same subject of a notification could be in different categories. For example, PPD as a subject could be classified as non-compliant, unauthorized, or inappropriate level depending on the issue of the reported product. Reports related to microbiological and heavy metal contamination were not further stratified (based on heavy metal type or bacterial/fungal taxon). Microbial contamination was the third most common (12%) quality issue in the examined products. Reports related to heavy metal contamination (lead and mercury) accounted for 5% of all reports. Additionally, the use of (elemental) heavy metals in cosmetic products is completely banned in the EU, while there is a tolerable limit for microbiological contamination.

In the examined products, aerobic mesophilic bacteria (*Enterobacter gergoviae, Pseudomonas aeruginosa, Enterobacter cloacae, Citrobacter freundii, Klebsiella pneumoniae ssp. pneumoniae, Pseudomonas putida, Pseudomonas Aeruginosa, Enterobacter cloacae, Enterobacter gergoviae*) were present. However, mold and fungal contaminations were also noted. A previous review of the EU Safety Gate focused on overpreserved and microbiologically contaminated products, and examined each product category from January 2008 to February 26, 2014. In the review, 11.76% of the products were recalled due to microbial contamination (80). This rate is consistent with that obtained from our results (12%). Overall, this quality problem was most commonly detected (within product types containing >7 reports/category) in dental hygiene products (46%) and hair products (17%).

Quality standards state that the concentration of aerobic mesophilic bacteria in cosmetic products intended for children aged under 3 years old and products intended for use on the eye's mucous membrane should not exceed 100 colony-forming unit/g or ml. Other products should not contain >1,000 colony-forming unit/g or ml (81). Higher-risk species, such as *Escherichia coli, Pseudomonas aeruginosa, Staphylococcus aureus*, and *Candida albicans* must not be found in 0.5 g or 0.1 ml of the first product category and in 0.1 g or 0.1 ml of any other product category (80, 82). Although preservatives can be used to prevent this type of quality issue, these compounds can also cause skin sensitization and other health problems (80).

## 5 Conclusions

According to a study, the average adult uses nine cosmetic products daily, while 25% of women use approximately 15 products on a daily basis. This can contribute to an increasing number of adverse events, such as allergies or contact dermatitis (83, 84). The average annual growth rate for the entire sector is expected to be between 6% and 8%, and the natural product sector is expected to grow at a similar rate. However, there are also growth forecasts of >20% (85–91). The use of botanical preparations increased visibly in 2018, in an analysis comparing the years 2011 and 2018 that focused mostly on naturally derived anti-aging products which suggesting a growing trend (92).

Using products that are thought to be natural may give a false sense of security, as they may also contain substances that are unsafe to health. Relatively little attention has been paid to the problems highlighted with greenwashed products. However, if a serious side effect develops after using a cosmetic product, health professionals and consumers may suspect a serious quality problem, in which case the use of the product should be suspended.

The majority of the reports we reviewed in this study identified a serious chemical risk. Skin-lightening products (even natural mimicking products) represented a wide variety of public health threats. The use of clobetasol and hydroquinone, may lead to severe skin damage and other adverse toxicological effects (28, 40, 46).

The high number of BMHCA-related reports might be explained by the fact that it has only recently been banned. However, this compound requires special attention from the authorities, due to its harmful effects on the reproductive system (31). Allergic contact dermatitis may arise from the inappropriate use of PPD in hair products, as well as from preservatives such as MCI/MI (15, 68).

Although most of the products with quality problems originate outside the EU, the notifications were made by EU Member States. This observation suggests that the presence of hazardous chemicals in products is a global problem that is receiving increasing attention in Europe. The Safety Gate system and proper risk communication, as well as more effective control of manufacturers and distributors by the authorities, can be effective tools to reduce the number of such incidents. Consumers can reduce their exposure to risk by self-educating, checking product ingredients, and choosing certified products and trusted manufacturers. It is the responsibility of public authorities and professionals to ensure that consumers have access to information that can form the basis of responsible choices.
