# Supplementary material for: Potential side effects of toxic and restricted substances found in cosmetics declared to be of natural origin
Source: Front Public Health. 2026 May 20;14:1801090. doi: 10.3389/fpubh.2026.1801090 (PMC13230110; doi:10.3389/fpubh.2026.1801090)
Supplement: Supplementary file 1 [file Data_Sheet_1.docx]

Supplementary Material

**Table S1.** Notifications in the EU Safety Gate from 2005-2023 allegedly natural products/all reports.

|  | Natural | All |
| --- | --- | --- |
| **2023** | 120 | 1098 |
| **2022** | 22 | 212 |
| **2021** | 9 | 52 |
| **2020** | 14 | 94 |
| **2019** | 11 | 103 |
| **2018** | 12 | 107 |
| **2017** | 6 | 64 |
| **2016** | 16 | 88 |
| **2015** | 3 | 47 |
| **2014** | 12 | 73 |
| **2013** | 14 | 92 |
| **2012** | 1 | 75 |
| **2011** | 10 | 104 |
| **2010** | 15 | 66 |
| **2009** | 9 | 86 |
| **2008** | 8 | 55 |
| **2007** | 7 | 81 |
| **2006** | 0 | 46 |
| **2005** | 0 | 18 |
| **Sum** | 289 | 2561 |

**Table S2.** Frequency of the substances within 324 substances/289 reports.

| Coumpound | N |
| --- | --- |
| **D-Limonene** | 1 |
| **Kojic acid** | 3 |
| ***o*-aminophenol** | 4 |
| **2-Methyl-3(2H)-isothiazolon** | 1 |
| **2-phenoxyethanol** | 2 |
| **5-Chlor-2-methyl-3 (2H)-isothiazolon** | 1 |
| **Amyl cinnamal** | 1 |
| **Barium peroxide** | 2 |
| **Benzoyl Peroxid** | 1 |
| **Benzyl alcohol** | 2 |
| **Benzyl cinnamate** | 1 |
| **Biofilm** | 1 |
| **Bishydroxyethyl biscetyl malonamide** | 3 |
| **BMHCA** | 125 |
| **Boron** | 1 |
| **Butyl paraben (Butyl 4-hydroxybenzoate)** | 1 |
| **Camphor** | 1 |
| **Carbamide peroxide** | 1 |
| **Cetrimide (mytrimonium bromide)** | 1 |
| **Cinnamyl alcohol** | 1 |
| **Citral** | 1 |
| **Clobetasol** | 13 |
| **Colorant (Pigment)** | 9 |
| **DEG** | 2 |
| **Eugenol** | 1 |
| **Formaldehyde** | 9 |
| **Geraniol** | 1 |
| **Heavy metal** | 16 |
| **Hexyl cinnamal** | 1 |
| **Hydroquinone** | 16 |
| **Hydroxyisohexyl 3-cyclohexene carboxaldehyde** | 2 |
| **IPBC (Iodopropynyl butylcarbamate ,CAS 55406-53-6)** | 1 |
| **Isobutylparaben** | 1 |
| **Isoeugenol** | 1 |
| **Limonene** | 1 |
| **Linalool** | 1 |
| **MCI** | 9 |
| **Methyldibromo glutaronitrile** | 2 |
| **MI** | 13 |
| **Microbial contamination** | 38 |
| **N-nitrosodiethanolamine** | 3 |
| **Not compliant** | 7 |
| **p-aminophenol** | 1 |
| **PPD** | 14 |
| **Pyrogallol** | 1 |
| **Salicylic acid** | 1 |
| **Sodium hydroxide** | 1 |
| **Sodium lauryl sulphate** | 1 |
| **Sodium perborate** | 3 |
| **Total** | 324 |

**Table S3.** Product types of the reported 289 products.

| Product type | N |
| --- | --- |
| **Aftershave** | 3 |
| **Baby all-over wash** | 2 |
| **Baby Wipes** | 1 |
| **Black cosmetic clay** | 1 |
| **Cream** | 34 |
| **Deodorant** | 6 |
| **Detergent** | 1 |
| **Eye cream** | 2 |
| **Face cream** | 5 |
| **Face mask** | 4 |
| **Facial toner** | 1 |
| **Foot balm** | 1 |
| **Hair product** | 82 |
| **Make-up** | 4 |
| **Make-up remover** | 4 |
| **Massage cream** | 3 |
| **Perfume** | 61 |
| **Peeling cream** | 3 |
| **Showering product** | 19 |
| **Skin lightening** | 37 |
| **Sunscreen** | 7 |
| **Tooth hygiene** | 8 |
| **Total** | 289 |

**Table S4.** Countries of origin and countries of submission of contaminated products.

| **Country of origin** | **N** | **Alert submitted by** | **N** |
| --- | --- | --- | --- |
| **Austria** | 3 | **Austria** | 7 |
| **Australia** | 1 | **Belgium** | 1 |
| **Belarus** | 1 | **Croatia** | 1 |
| **Belgium** | 9 | **Czechia** | 19 |
| **Brazil** | 5 | **Denmark** | 1 |
| **Bulgaria** | 1 | **Estonia** | 3 |
| **Czechia** | 8 | **Finland** | 13 |
| **Denmark** | 2 | **France** | 16 |
| **France** | 11 | **Germany** | 26 |
| **Germany** | 8 | **Hungary** | 55 |
| **Hong Kong** | 2 | **Ireland** | 14 |
| **Hungary** | 8 | **Italy** | 57 |
| **India** | 32 | **Latvia** | 1 |
| **Italy** | 36 | **Lithuania** | 14 |
| **Ivory Coast** | 15 | **Norway** | 8 |
| **Lao People's Democratic Republic** | 1 | **Poland** | 1 |
| **Lithuania** | 4 | **Portugal** | 12 |
| **Mexico** | 1 | **Romania** | 4 |
| **Morocco** | 11 | **Slovakia** | 7 |
| **Pakistan** | 12 | **Spain** | 2 |
| **People's Republic of China** | 11 | **Sweden** | 14 |
| **Philippines** | 1 | **The Netherlands** | 6 |
| **Poland** | 20 | **United Kingdom** | 7 |
| **Republic of Korea** | 1 | **NA** | NA |
| **Russian Federation** | 7 | **NA** | NA |
| **Senegal** | 1 | **NA** | NA |
| **South Africa** | 1 | **NA** | NA |
| **Spain** | 16 | **NA** | NA |
| **Switzerland** | 1 | **NA** | NA |
| **The Netherlands** | 3 | **NA** | NA |
| **Türkiye** | 10 | **NA** | NA |
| **United Arab Emirates** | 3 | **NA** | NA |
| **United Kingdom** | 10 | **NA** | NA |
| **United States** | 12 | **NA** | NA |
| **Unknown** | 21 | **NA** | NA |

* NA=Not Applicable

**Figure S1.** Risk categories created based on the quality issues of the substances.

**Figure S2.** Distribution of product categories within the examined 289 cosmetics with quality issues from 2007-2023.

*Other: Boron, Camphor, Methyldibromo glutaronitrile, Bishydroxyethyl biscetyl malonamide, N-Nitrosodiethanolamine, Barium peroxide, Butyl paraben (Butyl 4-hydroxybenzoate), Pyrogallol, Sodium perborate, 2-phenoxyethanol

**Figure S3.** Distribution of illegal substances or other quality issues within hair product category from 2007-2023 within 82 products/96 substances.

*Other aromatic compounds were the followings: D-Limonene, Citral, Geraniol, Benzyl alcohol, Cinnamyl alcohol, Linalool, Amyl cinnamal, Benzyl cinnamate, Hexyl cinnamal, Isoeugenol

**Figure S4.** Distribution of substances within perfume category 61 products/ 69 substances.

*Other: Hydroxyisohexyl 3-cyclohexene carboxaldehyde; Cetrimide (mytrimonium bromide); Benzoyl Peroxid; Methyldibromo glutaronitrile; Bishydroxyethyl biscetyl malonamide

**Figure S5.** Distribution of illegal substances or other quality issues within cream category 34 products/39 quality issues.

**Figure S6.** Distribution of illegal substances in skin lightening products 37 products/39 substances.

**Figure S7.** Distribution of illegal substances of other quality issues in shower products 19/19.

**Figure S8.** Distribution of substances and other quality issues within dental hygiene category 8 products/11 substances.

*Preservative: 5-Chlor-2-methyl-3 (2H)-isothiazolon and 2-Methyl-3(2H)-isothiazolon; IPBC=Iodopropynyl butylcarbamate (CAS 55406-53-6), Isobutylparaben **Other: Biofilm; Sodium lauryl sulphate and Sodium hydroxide in combination in eye lashes primer which can cause skin and eye irritation; Bishydroxyethyl biscetyl malonamide

**Figure S9.** Distribution of substances with quality issues in specific product category 48 products/ 51 quality issues.
